# Arginine Decarboxylase Gene (*adc*) Is Essential for *Vibrio anguillarum* Virulence and Physiological Phenotypes

**DOI:** 10.3390/microorganisms14030614

**Published:** 2026-03-09

**Authors:** Binghong Liu, Haichuan Li, Jinyuan Che, Baolong Bao

**Affiliations:** Key Laboratory of Exploration and Utilization of Aquatic Genetic Resources, Ministry of Education, National Demonstration Center for Experimental Fisheries Science Education, Shanghai Ocean University, Shanghai 201306, China; m230100053@st.shou.edu.cn (B.L.); 15380686273@163.com (H.L.)

**Keywords:** *V. anguillarum*, arginine, hemolysis, *adc*, virulence

## Abstract

*Vibrio anguillarum* is a major pathogenic bacterium causing vibriosis in aquatic animals, leading to substantial economic losses in the global aquaculture industry. Previous studies have indicated that L-arginine modulates the virulence of the pathogen, but the underlying molecular mechanisms remain elusive. The present study aimed to clarify the regulatory role of L-arginine metabolism in *V. anguillarum* virulence. We first evaluated the effects of L-arginine and its major metabolites (agmatine, putrescine, spermine) on the hemolytic activity of *V. anguillarum*. Results showed that L-arginine and its metabolites regulated hemolytic activity in a concentration-dependent biphasic manner, with agmatine exerting the most potent promoting effect. To identify the critical metabolic branch involved, four isogenic mutants were constructed targeting key genes in arginine metabolism (*adc*, *astA*, *astD*). Phenotypic analysis revealed that only the *adc* deletion mutant (Δ*adc*) exhibited near-complete loss of hemolytic activity, which was dose-dependently restored by supplementation with agmatine, putrescine, or spermine. Transcriptomic analysis identified 704 significantly differentially expressed genes (DEGs) between Δ*adc* and WT strains, with downregulated DEGs enriched in virulence-associated pathways. Key hemolysin and secretion system genes were validated to be downregulated in ∆*adc* by quantitative real-time PCR (qRT-PCR). Additionally, Δ*adc* displayed attenuated anti-phagocytic ability in Tetrahymena co-culture assays, impaired biofilm formation, and increased susceptibility to multiple classes of antibiotics. Collectively, our findings demonstrate that L-arginine modulates *V. anguillarum* hemolysis and overall virulence through the *adc*-mediated agmatine biosynthesis branch. This study fills the knowledge gap in the regulatory mechanism of L-arginine on *V. anguillarum* virulence and provides a potential target for the control of vibriosis in aquaculture.

## 1. Introduction

*Vibrio anguillarum* is a major bacterial pathogen in aquaculture and poses a serious threat to the global aquaculture industry. Infections caused by *V. anguillarum* can result in large-scale mortality of fish and crustaceans, leading to substantial economic losses worldwide [1,2]. The virulence of *V. anguillarum* is multifactorial and involves a broad array of virulence genes and determinants, including flagella, biofilm formation, iron acquisition systems, lipopolysaccharides (LPSs), and various extracellular products with proteolytic or hemolytic activities. Together, these factors facilitate bacterial colonization, infection, and disease progression within the host [3,4,5]. For instance, biofilm formation enhances bacterial tolerance to host immune defenses and antibiotic treatment, rendering infections more difficult to eradicate and often resulting in chronic infections and therapeutic failure

*V. anguillarum* is the etiological agent of vibriosis, an acute to subacute hemorrhagic septicemia that has been reported in a broad range of marine, brackish-water, and freshwater finfish. Susceptible and economically important hosts include salmonids (e.g., Atlantic salmon, rainbow trout), sea bass, turbot, striped bass, cod, eel, and red sea bream, among others, and outbreaks frequently cause rapid high mortality and major economic losses in both hatchery and grow-out systems [3,6].

In recent years, amino acid metabolism, particularly arginine (L-arginine) metabolism, has attracted increasing attention for its role in the regulation of bacterial virulence [7,8,9]. Arginine is a multifunctional amino acid that not only serves as a fundamental building block for protein synthesis but also plays crucial roles in microbial metabolism, survival, interspecies communication, and the expression of virulence traits [8,10]. Accumulating evidence indicates that arginine can significantly influence the pathogenic potential of a wide range of bacterial species [7,9].

Specifically, arginine-derived metabolites, such as the polyamines putrescine and agmatine, have been shown to be closely associated with bacterial virulence. Previous studies have demonstrated that microorganisms are capable of producing bioactive polyamines through complex biosynthetic pathways, and these molecules exert profound effects on host physiology and immune responses [11,12]. In *Streptococcus pneumoniae*, the SP_0916 gene encodes an arginine decarboxylase responsible for the production of agmatine, which is essential for capsular polysaccharide biosynthesis. The capsule is a critical virulence factor that enables *S. pneumoniae* to evade host immune surveillance [13]. Moreover, in *Vibrio fluvialis*, the arginine deiminase (ADI) pathway, under the regulation of ArgR, promotes bacterial survival under acidic conditions, highlighting the importance of arginine metabolism in bacterial environmental adaptation and virulence [14]. Notably, bacterial infection can also induce enhanced metabolic flux from arginine to spermidine in host cells, thereby suppressing NLRP3 inflammasome activation, further underscoring the pivotal role of arginine metabolism in host–pathogen interactions [15].

Despite the well-documented involvement of arginine and its metabolites in virulence regulation across diverse bacterial pathogens in humans, the role of arginine in *V. anguillarum* remains poorly understood. In particular, how arginine modulates its metabolic network to influence virulence and pathogenicity in *V. anguillarum* has not yet been systematically investigated. In aquaculture, arginine is routinely supplemented as a functional amino acid to support growth and immune competence. In parallel, the concept of prebiotics has expanded beyond non-digestible carbohydrates; ISAPP defines a prebiotic as “a substrate that is selectively utilized by host microorganisms conferring a health benefit” [16]. Emerging evidence suggests that some amino acids can act as “aminobiotics” with prebiotic-like effects by shaping the gut microbiota and its metabolites [17], and dietary L-arginine has been reported to alter intestinal microbiota profiles in fish (e.g., *Nile tilapia*) [18]. Given the increasing interest in prebiotics as antibiotic-sparing feed additives in aquaculture [19], it is important to clarify whether arginine supplementation could also influence opportunistic pathogens such as *V. anguillarum*, either directly through bacterial arginine metabolism or indirectly via microbiome-mediated interactions. Current knowledge gaps include the lack of mechanistic insight into the arginine metabolic network of *V. anguillarum* and its direct linkage to virulence-associated phenotypes, such as hemolysis and biofilm formation. Notably, the specific function and regulatory mechanisms of ADC, a key enzyme in arginine metabolism, during *V. anguillarum* pathogenesis remain largely unexplored.

This study aimed to systematically explore the regulatory role of L-arg and its metabolites in the hemolytic activity of *V. anguillarum* and clarify the underlying molecular mechanisms. We first investigated the effects of L-arg and its downstream metabolites (agmatine, putrescine, spermine) on the hemolytic activity of wild-type *V. anguillarum*. Then, we constructed four isogenic mutants targeting key genes in L-arg metabolism (∆*adc*, ∆*astA*, ∆*astD*, ∆*astA*∆*astD*) to identify the critical metabolic branch involved in hemolysis regulation. Additionally, we performed metabolite supplementation assays, transcriptomic analysis, and phenotypic validation to elucidate the regulatory role of the *adc* gene in *V. anguillarum* virulence. This study is the first to report the concentration-dependent biphasic regulation of *V. anguillarum* hemolysis by L-arg and its metabolites, providing novel insights into the molecular mechanisms of L-arg-mediated virulence regulation in aquatic pathogenic vibrios and a potential target for the prevention and control of *V. anguillarum*-associated vibriosis.

## 2. Materials and Methods

### 2.1. Strains, Media, and Experimental Animals

All bacterial strains and plasmids employed in this study are summarized in Table 1. The gene deletion mutant was generated and characterized in a *Vibrio anguillarum* background strain that was originally isolated from diseased *Larimichthys crocea* and is maintained in our laboratory collection. This strain was obtained previously from routine diagnostic samples and stored in our laboratory collection; no new animal sampling or bacterial isolation was performed and no identifiable patient data were involved, so ethics approval was not required under Shanghai Ocean University guidelines. Both the wild-type strain and its derivative mutants were routinely propagated in Luria–Bertani (LB) medium supplemented with 1% (*w*/*v*) NaCl at 30 °C with agitation at 150 rpm. For virulence assays, Tetrahymena, kindly provided by Prof. Shan Gao (Ocean University of China), was used as the infection model organism. Tetrahymena cultures were maintained in sterile SPP medium containing 2% proteose peptone, 0.1% yeast extract, 0.2% glucose, and 0.003% sequestrene, and incubated at 30 °C.

### 2.2. Construction of the adc, astA, astD, astAastD Deletion Mutant Strain

Four *adc*, *astA*, *astD*, *astAastD* gene deletion mutant strains (∆*adc*, ∆*astA*, ∆*astD*, ∆*astAastD*) were constructed via homologous recombination [20]. The primers used are listed in Table 2. The upstream and downstream flanking regions of *adc*, *astA*, *astD*, *astAastD* were amplified using the primers P1/P2 and P3/P4, respectively. These fragments were then ligated into the linearized suicide plasmid pSR47S, which had been digested with *Sac* I and *Sal* I, using the ClonExpress II One Step Cloning Kit (C112-01, Vazyme, Nanjing, China). The resulting plasmid was transformed into CC118 λpir, then conjugated into the wild-type (WT) strain. Mutants were selected on LB agar with kanamycin (Kan) and ampicillin (Amp), and screened on LB agar with 10% sucrose. Mutation was confirmed by PCR and sequencing using the primers T1/T2. Growth characteristics were evaluated by incubating cultures at 30 °C with shaking at 150 rpm until OD_600_ reached 1.0. The cultures were then diluted 1:100 and grown in a temperature-controlled incubator, with samples collected hourly.

### 2.3. Biofilm Formation

Biofilm formation ability was evaluated via a modified protocol. Briefly, 20 µL of overnight cultures of the wild-type (WT), ∆*adc*, ∆*astA*, ∆*astD*, and ∆*astAastD* strains were diluted 1:100 into fresh LB medium in 96-well plates, followed by incubation at 30 °C for 48 h. After incubation, the biofilms were gently washed to remove planktonic bacteria, stained with 0.1% crystal violet solution for 10 min, thoroughly rinsed with sterile water to eliminate excess stain, and then allowed to air dry. Subsequently, 100 µL of 95% ethanol was added to each well to dissolve the bound crystal violet, and the absorbance at 595 nm was measured using a microplate reader (Agilent, Cheadle, UK). All experiments were conducted in triplicate.

### 2.4. Hemolytic Activity Assay

All bacterial strains were grown overnight, after which 500 μL of bacterial culture was mixed with 50 μL of sterile, defibrinated sheep blood. L-arginine and its metabolic products, including agmatine, putrescine, and spermidine, were added to the mixtures at final concentrations of 0.625, 1.25, 2.5, 5, 50, 500, 5000, and 50,000 μM to generate hemolysis curves. The samples were incubated at 30 °C with shaking at 150 rpm for 3 h. Following incubation, the mixtures were centrifuged at 6000× *g* for 2 min, and 100 μL of the resulting supernatant was transferred to a 96-well microtiter plate. Absorbance was measured at 540 nm (OD_540_) using a microplate reader. Blank controls containing sterile, defibrinated sheep blood and the corresponding additives without bacterial inoculation were included, and background absorbance was subtracted accordingly. Each experiment was performed in triplicate. The hemolysis rate (%) was calculated according to the formula hemolysis rate (%) = [(A_540_,sample − A_540_,blank)/(A_540_,total − A_540_,blank)] × 100, where A_540_, sample represents the absorbance of samples incubated with bacterial culture and the indicated compounds; A_540_,blank represents the absorbance of sterile, defibrinated sheep blood with the corresponding additives but without bacterial inoculation; and A_540_,total represents the absorbance of sheep blood subjected to complete hemolysis.

To further evaluate hemolytic activity under solid conditions, bacterial cultures grown overnight were adjusted to the same cell density (OD_600_ = 1.0) using fresh LB medium. Aliquots (2 μL) of the standardized suspensions were carefully spotted onto Columbia blood agar plates supplemented with 5% (*v*/*v*) defibrinated sheep blood. The inoculated plates were incubated at 30 °C for 24 h. Hemolytic activity was assessed by visually examining the formation of transparent hemolytic zones surrounding the bacterial colonies. The diameters of hemolysis zones were measured using ImageJ 1.53, and hemolytic capacity was expressed as the mean zone diameter from three independent biological replicates.

### 2.5. Determination of Antibiotic Susceptibility

Antibiotic susceptibility of the strains was evaluated using kanamycin, 3ampicillin, spectinomycin, gentamicin, streptomycin, chloramphenicol, Rifampicin, clarithromycin, tobramycin and nalidixic acid. For each antibiotic, the half-maximal inhibitory concentration (MIC) was determined based on gradient treatments of the wild-type (WT) strain. Initially, all antibiotics were tested over a concentration range of 5–50 μg/mL with increments of 5 μg/mL. For kanamycin, gentamicin, chloramphenicol, rifampicin, and clarithromycin, tobramycin and tetracycline whose minimal inhibitory concentrations (MICs) were below 5 μg/mL, MIC values were further determined using a refined concentration gradient of 0.5–5 μg/mL with 0.5 μg/mL increments. In contrast, for ampicillin, whose MIC exceeded 50 μg/mL, an expanded concentration range of 55–155 μg/mL with 10 μg/mL increments was applied to determine the MIC. Overnight cultures of the WT and knockout strains were inoculated at 1% (*v*/*v*) into 96-well plates containing 200 μL of LB medium supplemented with the corresponding antibiotic concentrations. Cultures were incubated at 30 °C for 12 h, after which optical density was measured at 600 nm (OD_600_). All experiments were performed in triplicate.

### 2.6. Assessment of Strains’ Virulence Using Tetrahymena Model

The virulence of the ∆*adc* mutant was evaluated with a Tetrahymena infection model by measuring the relative survival of both bacteria and Tetrahymena after co-culture, as previously reported [21]. Briefly, Tetrahymena was grown in sterile SPP medium at 28 °C for 48 h, starting with an initial density of 10^3^ cells/mL. The cells were then collected by centrifugation at 2000× *g* for 10 min at 10 °C, washed twice with sterile SPP medium, and adjusted to a concentration of 1 × 10^5^ cells/mL.

Each bacterial strain was cultured for 12 h, harvested, washed twice with SPP medium, and resuspended to 3 × 10^9^ CFU/mL. Co-cultures were prepared by mixing *V. anguillarum* (3 × 10^9^ CFU/mL) with Tetrahymena (1 × 10^5^ cells/mL) at a ratio of 5000:1. The co-culture assays were divided into two experimental sets, including bacterial growth controls and Tetrahymena co-culture assays, resulting in a total of eight samples: WT, Δ*adc*, WT + ARG, and Δ*adc* + ARG for each set. For the co-culture assays, 100 μL of bacterial suspension was mixed with 100 μL of Tetrahymena culture, whereas for the growth control assays, 100 μL of bacterial suspension was mixed with 100 μL of LB medium. All mixtures were transferred into 96-well plates. For arginine-treated groups, L-arginine was added during the mixing process to a final concentration of 500 μM, followed by incubation at 30 °C for 5 h. followed by incubation at 30 °C for 5 h. Bacterial growth in the mixed suspension was monitored hourly by measuring the absorbance at 600 nm. Control groups contained bacterial strains mixed with an equal volume of SPP medium instead of Tetrahymena, and sterile SPP medium was used as the blank.

### 2.7. RNAseq and qRT-PCR Analysis

Wild-type (WT) and ∆*adc* mutant strains were cultured in LB medium at 30 °C overnight and then diluted 1:100 into fresh LB for 12 more hours. Six samples from each strain were collected by centrifugation, and total RNA was extracted using the RNAprep pure cell/bacteria kit (TianGen, Beijing, China). For RNA sequencing, six RNA samples from each strain were mixed proportionally to their molar mass and assessed with an Agilent 2100 bioanalyzer. Libraries were constructed with the NEBNext^®^ Ultra™ II Directional RNA Library Prep Kit (NEB, Ipswich, MA, USA) for paired-end 150 bp reads and sequenced on the Illumina NovaSeq 6000 platform by Novogene biotech (Beijing, China). Reads were aligned to the *V. anguillarum* 775 reference genome using Bowtie 2.3.4.3, and gene expression levels were quantified as RPKM. Differential expression analysis was performed with the edgeR package (3.24.3), setting a corrected *p*-value of 0.05 and a log2(Fold change) of 1 as thresholds for significance. DEGs related to key KEGG pathways were validated by qRT-PCR. Primers are shown in Table 3. Reverse transcription was done using a cDNA reverse transcription kit (Yeasen, Shanghai, China), and qRT-PCR was performed with an initial denaturation at 95 °C for 30 s, followed by 40 cycles of 95 °C for 10 s and 60 °C for 30 s. The relative expression of each gene in the mutant was quantified relative to WT strain after normalization to the 16S rRNA gene using the 2^−∆∆Ct^ method. The experiment was conducted in triplicate.

### 2.8. Statistical Analysis

All statistical analyses were performed using GraphPad Prism version 9 (GraphPad Software, San Diego, CA, USA). Differences among groups were evaluated by one-way or two-way analysis of variance (ANOVA), followed by Duncan’s multiple range post hoc test when appropriate. All experiments were conducted with at least three independent biological replicates, and data are presented as the mean ± standard deviation (SD). Statistical significance was defined as *p* < 0.05, with significance levels indicated as * *p* < 0.05, ** *p* < 0.01, *** *p* < 0.001, different uppercase letters indicate significant differences at *p* < 0.01.

## 3. Results

### 3.1. Effects of L-arginine and Its Metabolites on Hemolytic Activity of Vibrio Anguillarum

**Figure 1:**
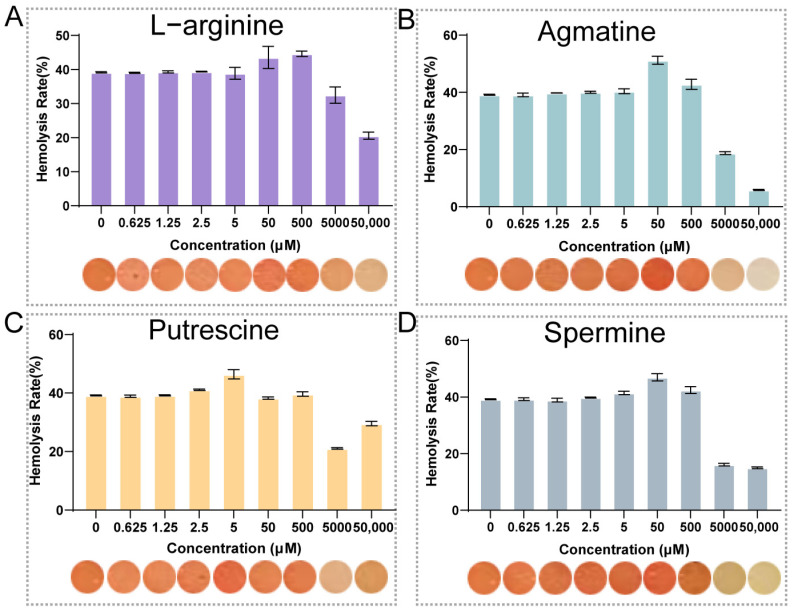
Concentration-dependent effects of L-arginine and its metabolites on *V. anguillarum* hemolytic activity. (**A**–**D**) Hemolysis rates of wild-type *V. anguillarum* following exposure to L-arginine (**A**), agmatine (**B**), putrescine (**C**), and spermine (**D**) at concentrations ranging from 0.625 to 5 × 10^4^ μM. Data are presented as mean ± SD (*n* = 3). Image of samples show the direct observation of hemolysis after centrifugation at 6000 rpm for 2 min (red is positively correlated with hemolysis rate).

To investigate the regulatory effects of L-arginine (ARG) and its metabolites, namely agmatine (AGM), putrescine (PUT), and spermine (SPE), on the hemolytic activity of the WT strain, we treated the strain with these substances at concentrations ranging from 0.625 to 5 × 10^4^ μM and determined the corresponding hemolysis rates. L-arginine (ARG) showed a stable hemolysis rate ranging from 39% to 40% at 0.625–5 μM, then increased to 46.4% (50 μM) and 45.4% (500 μM) at medium concentrations, and decreased to 32.5% and 21.2% at 5000 and 5 × 10^4^ μM, respectively (Figure 1A). Agmatine (AGM) displayed the most prominent promoting effect: its hemolysis rate remained at ~39–40% at 0.625–2.5 μM, peaked at 52.7% at 50 μM (the highest among all tested substances), and then dropped sharply to 19.2% and 6.1% at 5000 and 5 × 10^4^ μM, respectively. For putrescine (PUT), the hemolysis rate was sustained at ~39% at 0.625–2.5 μM, reached a peak of ~44.6% at 50 μM, and was significantly inhibited at concentrations ≥5000 μM. Similarly, spermine (SPE) exhibited a hemolysis rate ranging from 39% to 41% at 0.625–5 μM, peaked at 48.3% at 50 μM, and decreased to 16.6% and 15.1% at 5000 and 5 × 10^4^ μM, respectively.

In conclusion, L-arginine and its metabolites (AGM, PUT, SPE) all regulate the hemolytic activity of the WT strain in a concentration-dependent biphasic manner, with AGM showing the most significant promoting effect at 50 μM. These results elucidate the distinct regulatory roles of L-arginine and its metabolites in bacterial hemolytic activity, providing a clear basis for further mechanistic explorations.

### 3.2. Construction of Mutant Strains and Mechanistic Elucidation of Hemolytic Ability

**Figure 2:**
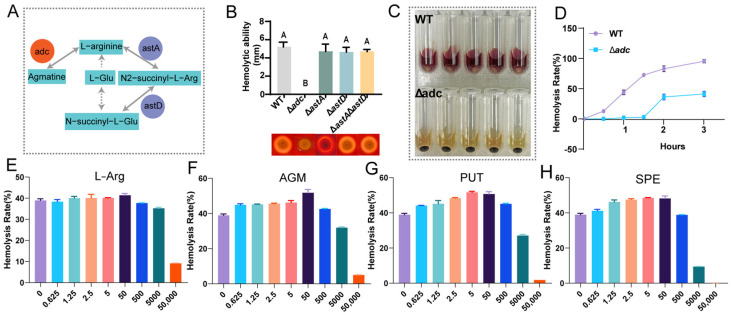
Genetic and metabolic dissection of arginine-dependent hemolysis in *Vibrio anguillarum*. (**A**) Schematic of arginine metabolic pathways in *V. anguillarum*. Key enzymes: *adc* (arginine decarboxylase), AstA (arginine N-succinyltransferase), AstD (succinylglutamic semialdehyde dehydrogenase). Metabolites: L-arginine, L-glutamate (L-Glu), agmatine, N_2_-succinyl-L-arginine, N(2)-succinyl-glutamate. (**B**) Hemolysis zone quantification (top) and representative images (bottom) of WT, ∆*astA*, ∆*astD*, and ∆*astA*∆*astD* on Columbia blood agar. Different uppercase letters indicate significant differences at *p* < 0.01 (one-way ANOVA with Tukey’s post hoc test). The data are presented as the mean ± SD (*n* = 3). (**C**) Representative visual assessment of liquid hemolysis for WT and ∆*adc*(red is positively correlated with hemolysis rate). (**D**) Kinetics of hemolysis for WT and ∆*adc* over 3 h. (**E**–**H**) Dose-dependent hemolysis restoration in ∆*adc* upon supplementation with L-arginine (**E**), agmatine (**F**), putrescine (**G**), and spermine (**H**) after 2 h incubation.

To dissect the mechanistic basis by which L-arginine and its metabolites modulate hemolysis in *Vibrio anguillarum*, we interrogated the metabolic network of L-arginine and its derivatives in this pathogen. The arginine metabolic pathway is evolutionarily conserved, and in *V. anguillarum*, arginine is channeled into two distinct metabolic branches: one catalyzed by arginine decarboxylase (*adc*, encoded by *adc*), directing arginine towards agmatine biosynthesis; the other initiated by arginine N-succinyltransferase (AstA, encoded by *astA*), with the resultant N_2_-succinyl-L-arginine further metabolized by succinylglutamic semialdehyde dehydrogenase (AstD, encoded by *astD*) to yield N(2)-succinyl-glutamate, a precursor for glutamate biosynthesis (Figure 2A). We generated a panel of isogenic mutants in *V. anguillarum* via homologous recombination: *adc* deletion mutant (∆*adc*), *astA* deletion mutant (∆*astA*), *astD* deletion mutant (∆*astD*), *astA*/*astD* double deletion mutant (∆*astA*∆*astD*), and deletion mutant (encodes argininosuccinate lyase, a rate-limiting enzyme in de novo arginine biosynthesis). To quantify hemolytic potential, we assessed hemolysis zone diameters on Columbia blood agar plates. Strikingly, only ∆*adc* exhibited a dramatic reduction in hemolytic activity, displaying near-abolished hemolysis. In contrast, ∆*astA*, ∆*astD*, and ∆*astA*∆*astD* showed hemolysis zones indistinguishable from the wild type (WT), indicating that disruption of the agmatine biosynthesis branch, but not the succinylative branch or de novo arginine biosynthesis, abrogates hemolysis (Figure 2B; *p* < 0.001, ns = not significant).

To corroborate these findings, we performed a time-course liquid hemolysis assay. Consistent with plate-based assays, ∆*adc* failed to induce significant hemolysis within the first 2 h of incubation with sheep erythrocytes, whereas WT achieved nearly complete erythrocyte lysis. Notably, ∆*adc* exhibited delayed hemolysis at later time points, with a hemolysis rate of ~36% at 3 h, whereas WT maintained a steadily increasing hemolysis rate, reaching near 100% at 3 h (Figure 2C,D).

To delineate the metabolite(s) mediating hemolysis in the *adc* pathway, we supplemented ∆*adc* cultures with graded concentrations of L-arginine (L-arg), agmatine (AGM), putrescine (PUT), and spermine (SPE), and measured hemolysis after 2 h. L-arg supplementation exerted a biphasic effect: low concentrations (≤5 μM) had no discernible impact, a moderate increase was observed at 50 μM, and higher concentrations (≥5 × 10^3^ μM) significantly inhibited hemolysis. In contrast, AGM, PUT, and SPE dose-dependently restored hemolytic activity in ∆*adc*. AGM at 0.625 μM elicited a 6% increase in hemolysis relative to the control without supplementation, and maximal recovery (51.8%, a 13% increase vs. control) was observed at 50 μM. Similarly, PUT and SPE showed concentration-dependent restoration, with peak hemolysis at 1 μM (51.6%) and 10 μM (48.5%), respectively (Figure 2E–H). These data collectively demonstrate that the agmatine biosynthesis branch, and its downstream polyamines, are pivotal for *V. anguillarum* hemolysis.

### 3.3. Imapct of the adc Mutant on V. anguillarum

**Figure 3:**
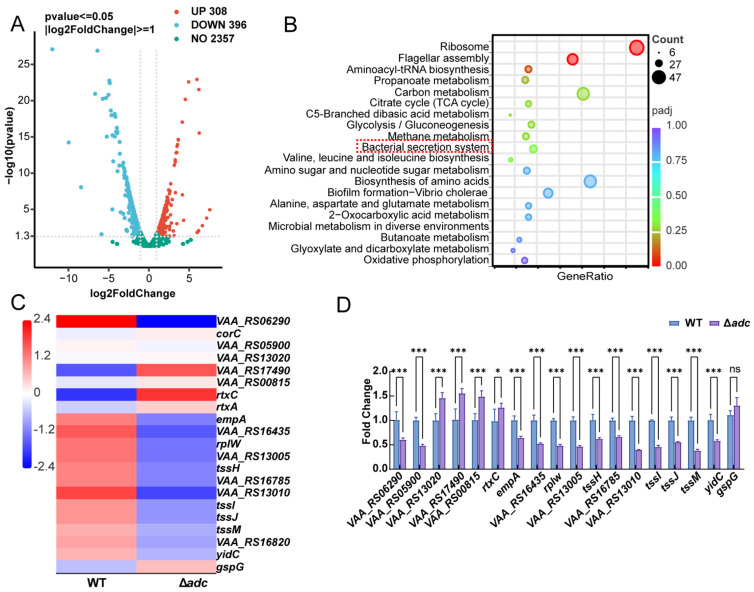
Transcriptomic analysis of *V. anguillarum* ∆*adc* mutant and wild-type (WT) strains. (**A**) Volcano plot showing differentially expressed genes (DEGs) between ∆*adc* mutant and WT strains (red dots: up-regulated genes; teal dots: down-regulated genes; black dots: genes with no significant change); DEGs were defined by |log_2_FoldChange| ≥ 1 and *p*-value ≤ 0.05. (**B**) Bubble plot of pathway enrichment analysis for down-regulated DEGs (Top 20). The size of bubbles represents gene count, and color represents *p*-value. Red indicates high expression. Green indicates low expression. (**C**) Hierarchical clustering of hemolysis and bacterial secretion system-related DEGs in ∆*adc* mutants compared with WT. (**D**) qPCR validation of expression levels of selected hemolysis and bacterial secretion system-related genes. The data are presented as the mean ± SD (*n* = 3). Columns have been marked with an asterisk (* *p* < 0.05; *** *p* < 0.001; ns: no significance).

Based on the above results, the deletion of *adc* critically impairs the hemolytic virulence of *V. anguillarum*. To explore the genome-wide regulatory effect of *adc* deficiency, we performed transcriptomic sequencing. A total of 704 significantly differentially expressed genes (DEGs) were identified between the ∆*adc* mutant and WT strains, including 308 up-regulated and 396 down-regulated genes (Figure 3A). KEGG pathway enrichment analysis of the down-regulated DEGs revealed the enrichment of multiple pathways associated with bacterial virulence and metabolism (Figure 3B). The most significantly enriched pathways included flagellar assembly; bacterial secretion system; biofilm formation—Vibrio cholerae; and glycolysis/gluconeogenesis, alongside basic metabolic pathways such as carbon metabolism and ribosome. These results indicate that *adc* deletion not only affects hemolysis-related virulence pathways but also broadly regulates bacterial secretion systems, motility, and energy metabolism. We further identified 21 DEGs closely associated with hemolysin synthesis and secretion, of which 2 were significantly up-regulated and 14 were significantly down-regulated. Hierarchical clustering heatmap analysis (Figure 3C) of these hemolysis and secretion system-related genes revealed that key hemolytic genes (e.g., *VAA_RS06290*(*vah3*), *empA*, *rtxC*) and Type VI secretion system (T6SS) genes (e.g., *tssH*, *tssM*, *tssJ*) were significantly down-regulated in the ∆*adc* mutant, with only a few genes showing up-regulation. This suggests that *adc* modulates hemolytic activity by regulating the expression of these virulence-associated genes. To confirm the transcriptomic findings, we performed qRT-PCR on a subset of key genes involved in hemolysis and the bacterial secretion system (Figure 3D). The results demonstrated that the expression of *vah3* (*VAA_RS06290*), *VAA_RS05900*, *empA*, *rtxC*, and other genes was significantly down-regulated in the ∆*adc* mutant, which was highly consistent with the transcriptomic data. This validation confirms that *adc* deletion negatively regulates the expression of these virulence-related genes.

### 3.4. Virulence Assessment of the ∆adc Mutant Using a Tetrahymena Co-Culture Model

**Figure 4:**
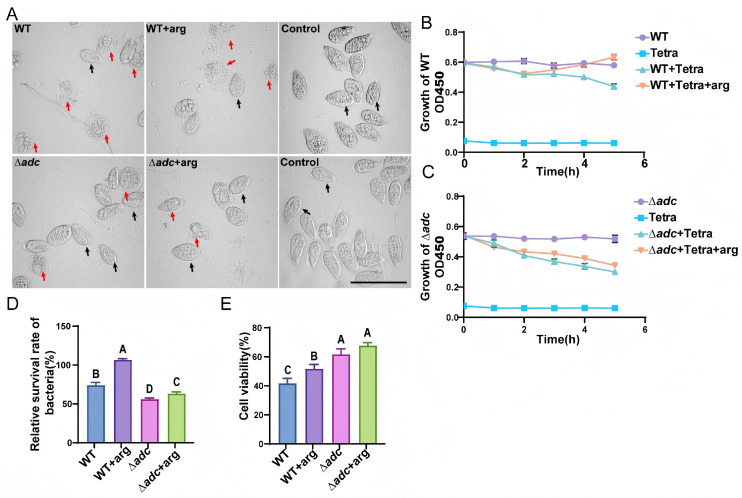
Virulence assessment of WT and ∆*adc* using the *Tetrahymena* model. (**A**) Morphological changes in Tetrahymena cells after co-cultivation with ∆*adc*, WT strains alone or treated with 500 μM arginine. Red arrow represents abnormal cell or died cell; black arrow represents normal cell. Scale bar: 100 μm. (**B**,**C**) Growth dynamics of ∆*adc* and WT strains co-cultured with or without *Tetrahymena* and effects of 500 μM arginine on the bacterial growth of ∆*adc* mutant strain co-cultured with Tetrahymena. Tetra represents *Tetrahymena*. (**D**) Relative survival was calculated as the OD_600_ of strains co-cultured with *Tetrahymena* divided by the OD_600_ of bacteria grown alone at the end of the experiment. Data are presented as mean ± SD from three independent measurements. Different uppercase letters indicate significant differences at *p* < 0.01. (**E**) Viability of *Tetrahymena* cells treated with lysates from the strains after 6 h, assessed using CCK8 reagents. Statistical comparisons were performed using one-way ANOVA analyses followed by a Dunnett’s multiple comparison test. Data are presented as the mean ± SD (*n* = 3). The significant difference in the results was analyzed. Different uppercase letters indicate significant differences at *p* < 0.01.

To further evaluate the virulence of the ∆*adc* mutant, a series of co-culture assays were performed with the ciliated protozoan Tetrahymena, a widely used model for investigating bacterial anti-phagocytic capacity and cytotoxicity. In the bacteria-free control group, Tetrahymena cells maintained normal cellular morphology and vigorous proliferative activity. Following co-incubation with the wild-type (WT) *V. anguillarum* strain, Tetrahymena exhibited severe morphological abnormalities, including marked cellular shrinkage and deformation, accompanied by significant growth suppression. In contrast, co-culture with the ∆*adc* mutant resulted in markedly alleviated cytotoxic effects: Tetrahymena displayed fewer aberrant cells and higher viable cell counts, indicative of attenuated virulence (Figure 4A). Bacterial growth dynamics during co-culture were monitored by measuring the optical density at 600 nm (OD_600_). After 6 h of co-incubation, the OD_600_ value of the ∆*adc* mutant was significantly lower than that of the WT strain (Figure 4B,C), demonstrating that the ∆*adc* mutant was phagocytosed at a substantially higher rate and exhibited impaired resistance to predation by Tetrahymena.

To further assess cytotoxicity, bacterial cell lysates of the WT and ∆*adc* strains were used to challenge Tetrahymena, and cell viability was quantified via the Cell Counting Kit-8 (CCK-8) assay. Tetrahymena cells treated with ∆*adc* lysates showed significantly higher viability than those exposed to WT lysates (Figure 4D), confirming that deletion of *adc* also reduced the cytotoxic potential of *V. anguillarum*. Collectively, these results demonstrate that loss of *adc* significantly compromises both the anti-phagocytic ability and cytotoxicity of *V. anguillarum*, leading to an obvious reduction in bacterial virulence.

### 3.5. Biofilm and Antimicrobial Susceptibility Defects in the ∆adc Mutant

**Figure 5:**
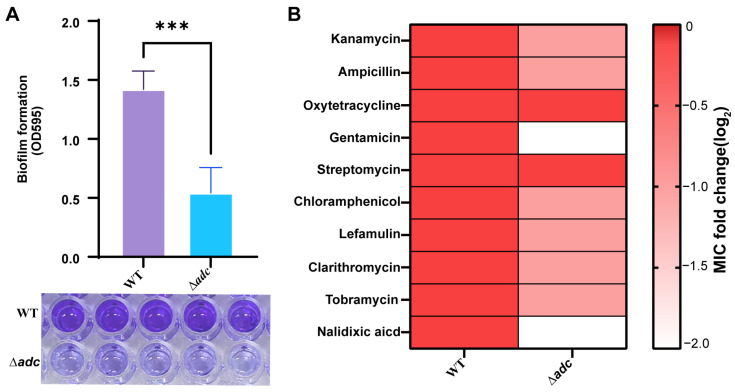
(**A**) Analysis of biofilm formation ability of WT and ∆*adc* strains. The data are presented as the mean ± SD (*n* = 5). Columns have been marked with an asterisk (*** *p* < 0.001); (**B**) Comparison of antibiotic susceptibility (MICs) to 10 antibiotics among WT and ∆*adc*. The fold change is calculated by log2 (the MIC of mutants/MIC of WT).

The ∆*adc* mutant exhibited a profound reduction in biofilm formation, with OD595 values decreasing from 1.4 ± 0.2 in the WT to 0.5 ± 0.1 in the mutant. Crystal violet staining confirmed impaired biofilm development (Figure 5A). To assess antimicrobial susceptibility, minimal inhibitory concentrations (MICs) of 10 clinically relevant antibiotics were determined, with results presented as log_2_ fold changes (∆*adc* MIC/WT MIC) (Figure 5B). The ∆*adc* mutant exhibited a 2-fold reduction in MICs for β-lactam antibiotics (cefotaxime, ampicillin), quinolones (nalidixic acid), macrolides (clarithromycin), and aminoglycosides (streptomycin, tobramycin), indicating increased susceptibility to these classes of antibiotics. In contrast, MICs for tetracycline, and chloramphenicol remained unchanged between the ∆*adc* mutant and WT strains (log_2_ fold change = 0). The heatmap in Figure 5B visually represents these differences, with red-to-white gradients indicating decreasing MIC ratios (∆*adc*/WT).

## 4. Discussion

This study investigated the regulatory function of arginine in *Vibrio anguillarum* by constructing a series of arginine metabolism-related deletion mutants (∆*adc*, ∆*astA*, ∆*astD*, ∆*astAastD*) and performing metabolite supplementation, transcriptomic sequencing and multiple phenotypic assays. Our results demonstrated that *adc* mediates the agmatine synthesis branch of L-arginine metabolism, and deletion of *adc* significantly abolished the hemolytic activity of *V. anguillarum*, downregulated the transcription of multiple virulence-related genes, reduced bacterial anti-phagocytic capacity and cytotoxicity, impaired biofilm formation, and increased susceptibility to multiple classes of antibiotics.

L-arginine (L-arg) exhibits remarkable metabolic and regulatory diversity across diverse organisms, being ubiquitous in all domains of life and playing a pivotal role in host–microbe interactions [8,9]. As such, L-arg metabolism is centrally important for numerous biological processes, as well as the crosstalk between mammals, microbes, and plants [8,22,23]. Beyond serving as a precursor for polyamine biosynthesis and a building block for protein synthesis, L-arg is critical for microbial growth, differentiation, and energy metabolism [8,22]. Consequently, the availability and metabolism of L-arg in microbes are tightly regulated by multiple anabolic and catabolic enzymatic pathways. Previous studies have demonstrated that exogenous L-arg supplementation induces virulence gene expression in enterohemorrhagic Escherichia coli (EHEC) in vitro, while arginine transport-deficient mutants (∆*artP*) show reduced virulence gene expression [6]. In 2023, Björn Nüse et al. further highlighted the enzymatic pathways of L-arg metabolism in microbes and mammalian cells, as well as their significant roles in immune function, luminal metabolism, colonization resistance, and the pathogenic mechanisms of intestinal microbes [7]. However, limited studies have addressed the effects of L-arg and its metabolism on bacterial virulence in aquatic pathogenic *vibrios*, particularly *Vibrio anguillarum*-a major pathogen threatening both marine and freshwater cultured animals. In this study, we are the first to report that L-arginine and its downstream metabolites (agmatine, putrescine, spermine) regulate the hemolytic activity of wild-type *V. anguillarum* in a concentration-dependent biphasic manner. Notably, although high concentrations of L-arg inhibit bacterial hemolysis and significantly reduce virulence, the natural concentration of L-arg in aquatic environments is generally very low. Thus, the inhibitory effect of high concentrations is physiologically irrelevant under natural conditions, and L-arg typically exerts a stimulatory effect at environmentally relevant low concentrations. Among the tested metabolites, agmatine exhibited the most potent stimulatory effect on hemolysis, further emphasizing its key role in this regulatory axis.

From a translational perspective, our findings highlight the need to validate arginine-based interventions in fish. Dietary arginine can benefit host growth/immune function and may exert prebiotic-like effects on gut microbiota [17,18,19]; however, our in vitro data indicate that low micromolar arginine and polyamines can enhance *V. anguillarum* hemolysis. Therefore, controlled fish challenge experiments and, ultimately, pond/net-pen field trials are required to determine the net outcome of arginine supplementation under realistic farming conditions (e.g., temperature/salinity, stocking density, background microbiota). Such studies should quantify survival, bacterial loads, and host immune/barrier responses, while monitoring gut microbiome and metabolite changes and testing dose/timing regimens to minimize any unintended promotion of pathogen virulence [24].

While a limited number of studies have reported that L-arg modulates bacterial virulence, the underlying mechanisms by which L-arg promotes virulence and regulates hemolysin secretion remain unclear in *V. anguillarum*. In this pathogen, L-arg metabolism primarily proceeds through two distinct pathways: one catalyzed by arginine decarboxylase (encoded by *adc*) that directs L-arg toward agmatine biosynthesis, and the other mediated by arginine N-succinyltransferase (encoded by *astA*) and succinylglutamate desuccinylase (encoded by *astD*) that metabolizes L-arg to L-glutamate. To dissect the mechanism by which L-arg influences virulence, we constructed four isogenic mutants targeting key genes in these pathways (∆*adc*, ∆*astA*, ∆*astD*, ∆*astA*∆*astD*). Phenotypic analysis revealed that only the ∆*adc* strain exhibited near-complete loss of hemolytic activity, whereas ∆*astA*, ∆*astD*, and ∆*astA*∆*astD* showed hemolytic phenotypes indistinguishable from the wild-type strain. Moreover, exogenous supplementation with agmatine, putrescine, or spermine dose-dependently restored the hemolytic activity of ∆*adc*, confirming that these polyamines are the critical effectors of the *adc*-mediated pathway.

Previous studies have confirmed that polyamines act as important signaling molecules regulating the virulence of pathogenic Vibrio species and other pathogens [12,25,26,27]. Our results further clarify that the *adc*-dominated agmatine biosynthesis branch—rather than the succinylative catabolic branch or de novo arginine synthesis pathway—serves as the key metabolic node for L-arginine-mediated regulation of *V. anguillarum* hemolysis. This finding supplements the regulatory mechanism of arginine metabolism on core virulence phenotypes in aquatic *vibrios*. The *adc* gene and its mediated arginine decarboxylation pathway have been widely studied in Gram-negative bacteria, primarily focusing on bacterial acid stress tolerance and polyamine synthesis (e.g., Escherichia coli, Vibrio cholerae, Pseudomonas aeruginosa) [1,2,3]. However, the specific role of *adc* in regulating the virulence and physiological phenotypes of *V. anguillarum*—a dominant aquatic pathogen with significant impacts on aquaculture—has not been systematically elucidated prior to this study. Our findings thus fill this knowledge gap, highlighting the species-specific regulatory function of *adc* in linking arginine metabolism to virulence in *V. anguillarum*.

Transcriptomic analysis showed that 704 differentially expressed genes were identified between the ∆*adc* mutant and the wild-type strain, and the down-regulated genes were significantly enriched in flagellar assembly, bacterial secretion system, biofilm formation and glycolysis/gluconeogenesis pathways. Key hemolysin genes such as *vah3*, *empA*, *rtxC* and Type VI secretion system (T6SS) genes including *tssH*, *tssM*, *tssJ* were significantly down-regulated, and the qRT-PCR results were highly consistent with the transcriptomic data. It has been documented that the virulence of pathogenic Vibrio is a complex trait regulated by multiple gene networks, and hemolysin, secretion system and flagellar system are usually co-regulated to jointly mediate bacterial adhesion, invasion and cytotoxicity [3,28]. The widespread down-regulation of virulence and metabolic pathways in the ∆*adc* mutant indicates that *adc* is not only a structural gene involved in arginine metabolism, but also a global regulatory factor affecting the physiological and pathogenic processes of *V. anguillarum*.

In addition, the ∆*adc* mutant exhibited reduced biofilm formation and lower MICs to several antibiotic classes. Importantly, these MIC shifts in an isogenic, laboratory-constructed mutant should not be interpreted as antimicrobial resistance (AMR) in the epidemiological sense; AMR is driven by genetic change and selection under antimicrobial exposure and is accelerated by misuse and overuse of antibiotics [29,30]. Instead, our data most likely reflect altered intrinsic susceptibility/tolerance linked to metabolic homeostasis, envelope permeability, and/or biofilm-associated protection [31,32]. Biofilms can promote environmental persistence and antimicrobial tolerance by restricting penetration, altering metabolic states, and enabling stress responses [31]. Intracellular amino acid metabolism and polyamines are also known to regulate biofilm maturation in Gram-negative bacteria [33,34]. Combined with transcriptomic data, the impaired biofilm formation in ∆*adc* may stem from metabolic imbalance induced by blocked arginine decarboxylation, though the specific polyamine-mediated mechanism requires further validation. Overall, the increased antibiotic susceptibility of ∆*adc* suggests that *adc*-dependent polyamine metabolism contributes to baseline stress tolerance and cell envelope homeostasis rather than acquired AMR.

In the *Tetrahymena* co-culture model, the ∆*adc* mutant displayed significantly reduced growth and diminished cytotoxicity of its cell lysates toward *Tetrahymena*, directly demonstrating that deletion of *adc* attenuates the anti-phagocytic ability and cytotoxicity of *V. anguillarum*—two key virulence traits underlying bacterial pathogenicity. As a ubiquitous predator in aquatic ecosystems, *Tetrahymena* exerts strong selective pressure on the virulence evolution of aquatic pathogenic bacteria, and bacterial strains with compromised anti-phagocytic capacity typically exhibit attenuated pathogenic potential in hosts [21,35]. Our results confirm that *adc* is essential for mediating *V. anguillarum*’s environmental adaptability and pathogenicity by regulating anti-phagocytic capacity. Notably, the attenuated virulence of ∆*adc* is not limited to anti-phagocytosis and cytotoxicity: the mutant also shows drastically reduced hemolytic activity, impaired biofilm formation, and increased susceptibility to multiple classes of antibiotics. Collectively, these multi-faceted virulence defects align with the notion that gene deletion strains with disrupted key metabolic pathways and attenuated virulence are promising candidates for attenuated live vaccines [15,20]. Thus, the ∆*adc* strain exhibits comprehensive virulence attenuation mediated by a core metabolic pathway, offering a novel and effective strategy for the control of vibriosis in aquaculture.

## 5. Conclusions

In conclusion, this study systematically clarified the pivotal regulatory role of arginine in *V. anguillarum* using a panel of arginine metabolism-related deletion mutants, with a focus on the *adc* deletion mutant (∆*adc*). Results showed that *adc* deletion abolished hemolytic activity, impaired anti-phagocytic ability and biofilm formation and increased susceptibility to multiple antibiotics, while exogenous agmatine, putrescine or spermine restored hemolysis. These findings align with studies in other Gram-negative bacteria, highlighting *adc*’s conserved role in linking arginine metabolism to bacterial virulence. Notably, *adc* deletion attenuated *V. anguillarum* pathogenicity by disrupting agmatine biosynthesis, suggesting ∆*adc* as a potential candidate for attenuated vaccines against vibriosis. However, the study was limited by the lack of long-term in vivo fish infection models and unclear downstream molecular targets of *adc*. Future studies should address these limitations to evaluate the long-term protective efficacy of ∆*adc* strains and clarify the detailed regulatory mechanisms of *adc*, facilitating the development of effective strategies to control *V. anguillarum* infections.

## Figures and Tables

**Table 1:** Strains and plasmids.

Strains	Genotype and Characteristics	Source
*V. anguillarum*	Cms, Kms, Ampr, Wild-type strain	Our lab
∆*adc(VAA_RS15135)*	*V. anguillarum* strain in-frame deletion in *adc*	This study
∆*astA(VAA_RS02805)*	*V. anguillarum* strain in-frame deletion in *astA*	This study
∆*astD(VAA_RS02810)*	*V. anguillarum* strain in-frame deletion in *astD*	This study
∆*astA*∆*astD*	*V. anguillarum* strain in-frame deletion in ∆*astA*∆*astD*	This study
*Escherichia coli*		
CC118	λpir lysogen of CC118 (∆(ara-leu) araD ∆lacX74galEgalKphoA20 thi-1rpsE rpoB argE (Am) recA1	Our lab
CC118/pHelper	CC118 λpir harboring plasmid pHelper	Our lab
Plasmids		
pSR47S	Bacterial allelic exchange vector with sacB, KanR	Our lab
pSR47S-∆*adc*	A 1204 bp fragment encompassing the upstream and downstream regions of the *adc* gene in pSR47S, KanR	This study
pSR47S-∆*astA*	A 1398 bp fragment encompassing the upstream and downstream regions of the *astA* gene in pSR47S, KanR	This study
pSR47S-∆*astD*	A 1253 bp fragment encompassing the upstream and downstream regions of the *astD* gene in pSR47S, KanR	This study
pSR47S-∆*astAastD*	A 1362 bp fragment encompassing the upstream and downstream regions of the *astAastD* gene in pSR47S, KanR	This study

**Table 2:** Sequences of PCR oligonucleotide primers.

Primer Name	Primer Sequence (5′ to 3′)	Purpose
*adc-P1*	AAAGGGAACAAAAGCTGGCTTCGGCGCATGATAGAACA	Creation of ∆*adc* deletion fusion fragment
*adc-P2*	CAGGCGTCAAGTCGTGATATAGGCTTGAGCGGTTATACCT	
*adc-P3*	ATATCACGACTTGACGCCTG	
*adc-P4*	TCGATCCTCTAGAGTCGAGGCCGATAAACGGCTAAATC	
*astA-P1*	AAAGGGAACAAAAGCTGGCCTTCGGCTGAGTTTGTCCAAG	Creation of ∆*astA* deletion fusion fragment
*astA-P2*	AGCTAACCATTCACCTGTAAATCGTAGTCCGACATTGCGATAG	
*astA-P3*	TTACAGGTGAATGGTTAGCTGGAC	
*astA-P4*	TCGATCCTCTAGAGTCGACAGCAAACTGGCGATGGAGT	
*astD-P1*	AAAGGGAACAAAAGCTGGGCTGAAATGCGTGGTGTGTC	Creation of ∆*astD* deletion fusion fragment
*astD-P2*	CTCCTTGTCTGTGCTCGGTTGTCCAGCTAACCATTCACCTGTAA	
*astD-P3*	AACCGAGCACAGACAAGGAG	
*astD-P4*	TCGATCCTCTAGAGTCGAGACTGCTCAAGCAGCACATCAG	
*astAastD-P1*	AAAGGGAACAAAAGCTGGCCTTCGGCTGAGTTTGTCCAAG	Creation of ∆*astAastD* deletion fusion fragment
*astAastD-P2*	CTCCTTGTCTGTGCTCGGTTATCGTAGTCCGACATTGCGATAG	
*astAastD-P3*	AACCGAGCACAGACAAGGAG	
*astAastD-P4*	TCGATCCTCTAGAGTCGGACTGCTCAAGCAGCACATCAG	
*adc-T1*	GTACCACGACATCGGCATCTG	Confirmation of deletion strain
*adc-T2*	ATGTGCGTATGCGCGTATCG	
*astA-T1*	CATCTCTGATCGCACTTGTGC	
*astA-T2*	AAGCTCGCCATACTGCTTAGAGA	
*astD-T1*	TTCTGATGATGGCGGAACACC	
*astD-T2*	CTTCCGTAAATTGCACCGGTAC	
*astAastD-T1*	CATCTCTGATCGCACTTGTGC	
*astAastD-T2*	CTTCCGTAAATTGCACCGGTAC	

Note: Complementary sites are underlined.

**Table 3:** Primers used for qRT-PCR.

Primer Name	Primer Sequence (5′ to 3′)	Tm (°C)	Amplicon Size (bp)
VAA_RS16785-f	CTGGTTTCAACTGCGTGGTG	58	220
VAA_RS16785-r	AAACGCGCACAGAATGTAGC	58	
VAA_RS00815-f	ACCACCAACAGCAAGTAGCA	58	206
VAA_RS00815-r	TTCGCACTCCTTTGGCGATA	58	
VAA_RS17490-f	ACCGTGTTGAACGTAGCACT	59	232
VAA_RS17490-r	ATGGTGAACTCCGTTGTCCC	59	
VAA_RS16435-f	GCGCTACTGGCATTGGAAAG	57	193
VAA_RS16435-r	GTACCGCAGCTGTAGTCTCC	58	
VAA_RS16790-f	TTGGTTATGGTGAGGGTGGC	59	276
VAA_RS16790-r	AAGCCGCAAGTTCTGGGTAA	59	
VAA_RS16775-f	TCCTGCTGATGCTACTGACG	58	154
VAA_RS16775-r	ACTTGAACCTCTATCGGCGAC	58	
VAA_RS08510-f	CAGTTGCGTTTCCACCACAG	59	239
VAA_RS08510-r	CCTCACGAATACTGGCGGAA	58	
VAA_RS06290-f	TACGCCATATGTCCACCACG	59	129
VAA_RS06290-r	GCGATTGGATCAACAGCCAG	59	
VAA_RS16810-f	TGGATTGGCGATGAATCGGT	59	203
VAA_RS16810-r	GTTGCAGTTGCGGTTGCTAA	57	
VAA_RS10665-f	CCGTGACACCGATGTGACTAT	59	251
VAA_RS10665-r	TGAAAGCGCTGGTACTGTGA	58	
VAA_RS08515-f	GCCGAAGCGTGGAAGAAATC	58	237
VAA_RS08515-r	GGACGAAGCTGAGGCCAATA	58	
VAA_RS10655-f	ACCAGCACCACGACGATATT	59	249
VAA_RS10655-r	ACAAGCCGCAGATCAGTTCA	59	
VAA_RS13010-f	GTTGACCAGAAGGCTGACCT	58	137
VAA_RS13010-r	CACTGCTGGTGCTCTGTCTA	57	
VAA_RS01050-f	TCCAGCAATGGCAAGTAGCA	59	125
VAA_RS01050-r	CGCATTGGTTACTGCTGGTG	58	
VAA_RS15875-f	GCTCAACCAGCGCTAAAGT	57	160
VAA_RS15875-r	TAGGTGGTTCCTGGTGTTGC	59	
VAA_RS13005-f	GGTAGACCAAGCCCTCTTCG	59	120
VAA_RS13005-r	TTGACGACTATGCCTTCGCA	57	
VAA_RS16150-f	AAGGTGTGGAATCGCTGTCT	59	152
VAA_RS16150-r	AAGGTCATCACGCAGCATCT	59	
VAA_RS05900-f	TTCTCATCCTCGCTGGTTGG	59	265
VAA_RS05900-r	GGCCAGTTGGCCAAATTCAA	58	
VAA_RS02910-f	CGACCTTGGCGTAGACCTT	59	163
VAA_RS02910-r	TGACTGCTGAGAAAGCGAAC	57	
VAA_RS14860-f	ACAGCGGTTGATCACAAGGT	59	251
VAA_RS14860-r	GTGCGCGGATCATGAACAAA	58	
VAA_RS14800-f	CGGTATTCGACATCGGTGGT	59	247
VAA_RS14800-r	GTTTTACTCGCCGCAGTCAC	58	
VAA_RS11670-f	GTGCCATGATTGGTGAAGGC	58	127
VAA_RS11670-r	GCGCTGTACACAACGTTACC	59	
VAA_RS00200-f	CACCCTCTTGACCATCAGCA	57	163
VAA_RS00200-r	CTAGTGACCAAGCCGTCGAA	58	
VAA_RS13020-f	TAGTCGACCGTATTGTGCCG	59	129
VAA_RS13020-r	CAATGTCGACGACTGAACGC	59	
VAA_RS06905-f	GTGAATGGCAGCAACGGATT	58	180
VAA_RS06905-r	TATGATCACCAGAGCGCCAG	59	
16s rRNA-F	TTAAGTAGACCGCCTGGGGA	58	185
16s rRNA-R	GCAGCACCTGTCTCAGAGTT	59	

## Data Availability

The data that support the findings of this study are available from the FigShare at 10.6084/m9.figshare.31220680 or the corresponding author upon reasonable request.
